# Stimulation of soluble guanylate cyclase improves donor organ function in rat heart transplantation

**DOI:** 10.1038/s41598-020-62156-y

**Published:** 2020-03-24

**Authors:** Kálmán Benke, Balázs Tamás Németh, Alex Ali Sayour, Klára Aliz Stark, Attila Oláh, Mihály Ruppert, Gábor Szabó, Sevil Korkmaz-Icöz, Eszter Mária Horváth, Rita Benkő, István Hartyánszky, Zoltán Szabolcs, Béla Merkely, Tamás Radovits

**Affiliations:** 1 Heart and Vascular Center, Semmelweis University, Budapest, Hungary; 2 Department of Cardiac Surgery, University of Heidelberg, Heidelberg, Germany; 3 Department of Physiology, Semmelweis University, Budapest, Hungary; 4 Department of Cardiac Surgery, University of Halle, Halle, Germany

**Keywords:** Cardiac regeneration, Heart failure

## Abstract

Heart transplantation remains the definitive therapy of end-stage heart failure. Ischemia-reperfusion injury occurring during transplantation is a primary determinant of long-term outcome of heart transplantation and primary graft insufficiency. Modification of the nitric oxide/soluble guanylate cyclase/cyclic guanosine monophosphate signaling pathway appears to be one of the most promising among the pharmacological interventional options. We aimed at characterizing the cardio-protective effects of the soluble guanylate cyclase stimulator riociguat in a rat model of heterotopic heart transplantation. Donor Lewis rats were treated orally with either riociguat or placebo for two days (n = 9) in each transplanted group and (n = 7) in donor groups. Following explantation, hearts were heterotopically transplanted. After one hour reperfusion, left ventricular pressure-volume relations and coronary blood flow were recorded. Molecular biological measurements and histological examination were also completed. Left ventricular contractility (systolic pressure: 117 ± 13 vs. 48 ± 5 mmHg, *p* < 0.001; dP/dt_max_: 2963 ± 221 vs. 1653 ± 159 mmHg/s, *p* < 0.001), active relaxation (dP/dt_min_: −2014 ± 305 vs. −1063 ± 177 mmHg/s, *p* = 0.02; all at 120 µl of left ventricular volume), and alteration of coronary blood flow standardized to heart weight (2.55 ± 0.32 vs. 1.67 ± 0.22 ml/min/g, *p* = 0.03) were markedly increased following preconditioning with riociguat. Myocardial apoptosis markers were also significantly reduced in the riociguat pretreated group as well as the antioxidant markers were elevated. Pharmacological preconditioning with riociguat decreases ischemia-reperfusion injury and improves donor organ function in our animal model of heart transplantation. Therefore, riociguat might be a potential cardioprotective agent.

## Introduction

Heart transplantation (HTX) is still the optimal procedure for patients with end-stage heart failure, when other medical or surgical interventions have failed. The primary graft failure (PGF) remains an important problem after heart transplantation; it is reported to have an incidence up to even 40%^1^. Ischemia/reperfusion (I/R) injury is a major determinant of PGF. The deleterious effects of I/R injury are mediated mainly by reactive oxygen (ROS) and nitrogen species (RNS) such as peroxynitrite^2^, production of which during ischemia is enhanced. This is followed by an extra burst of reactive species generation taking place at reperfusion. ROS and RNS tissue concentrations at any time are dependent on the balance of their production and elimination, the latter being actively regulated by antioxidant enzymes in the endogenous antioxidant system, such as superoxide dismutase (SOD) and catalase^3^. Therefore, in order to reduce I/R injury, reduction of the amount of ROS and RNS either by decreasing production or enhancing elimination is essential. A feasible option to achieve this goal during transplantation is pharmacological preconditioning the heart before explantation.

Oxidative and nitrosative stress-mediated injury leads to dysfunction of the nitric oxide (NO)/soluble guanylate cyclase (GC-1)/cyclic guanosine monophosphate (cGMP)/protein kinase G (PKG) pathway^4^. This pathway has anti-apoptotic and anti-inflammatory effects via regulation of endogenous antioxidant mechanisms, as well as regulation of cell adhesion molecules important in leukocyte invasion, respectively. Experimental data shows that either increasing cGMP production by administration of a GC-1 activator (e.g. cinaciguat) or stimulator (e.g. riociguat), or inhibiting cGMP degradation by blocking phosphodiesterase-5 (PDE5) using sildenafil, vardenafil or tadalafil, resulted in beneficial effects in various models of I/R injury and myocardial infarction^5^. Therefore, agents that boost cGMP synthesis via stimulating GC-1 might become important therapeutic options for the prevention of I/R injury associated with heart transplantation.

Drugs that are capable of either activating or stimulating GC-1 have been developed in the last decades. After a promising pre-clinical stage^6^, however, GC-1 activators as cardiovascular drugs have failed to impress in the clinical setting. Cinaciguat, the most potent GC-1 activator to date, has been shown to cause severe hypotension in patients with acute decompensated heart failure (HF)^7^, resulting in early termination of both clinical trials utilizing the drug. A more recent, possibly valuable therapeutic option for the treatment of I/R injury might be, due to its less pronounced hypotensive effect, the stimulation of GC-1. As the NO/GC-1/cGMP pathway is an important determinant of blood pressure - in cardiomyocyte specific deletion of NO-GC-1 knockout mice – a significant elevation was observed in both systolic and diastolic blood pressure values^8^. GC-1 stimulators have a dual mode of action, as they sensitize GC-1 to endogenous NO as well as directly stimulating reduced GC-1 in the absence of NO^9^. On the other hand, Tsai and colleagues firstly identified differential NO/heme-dependent and -independent activation properties of GC-1^10^. The first GC-1 stimulator agent to succeed in clinical trials was riociguat (Adempas, Bayer AG, Leverkusen, Germany)^11^. Although riociguat currently is accepted as a class II or III drug for the treatment of pulmonary arterial hypertension (PH) or chronic thromboembolic pulmonary hypertension, accumulating experimental and clinical data show its cardioprotective effect^12,13^. Therefore, our aim was to investigate the cardioprotective capabilities of stimulating the NO/GC-1/cGMP pathway using riociguat in a well-established rodent model of HTX^14–16^.

## Results

### Riociguat treatment increases tissue cGMP content, resulting in increased PKG signaling

**Figure 1:**
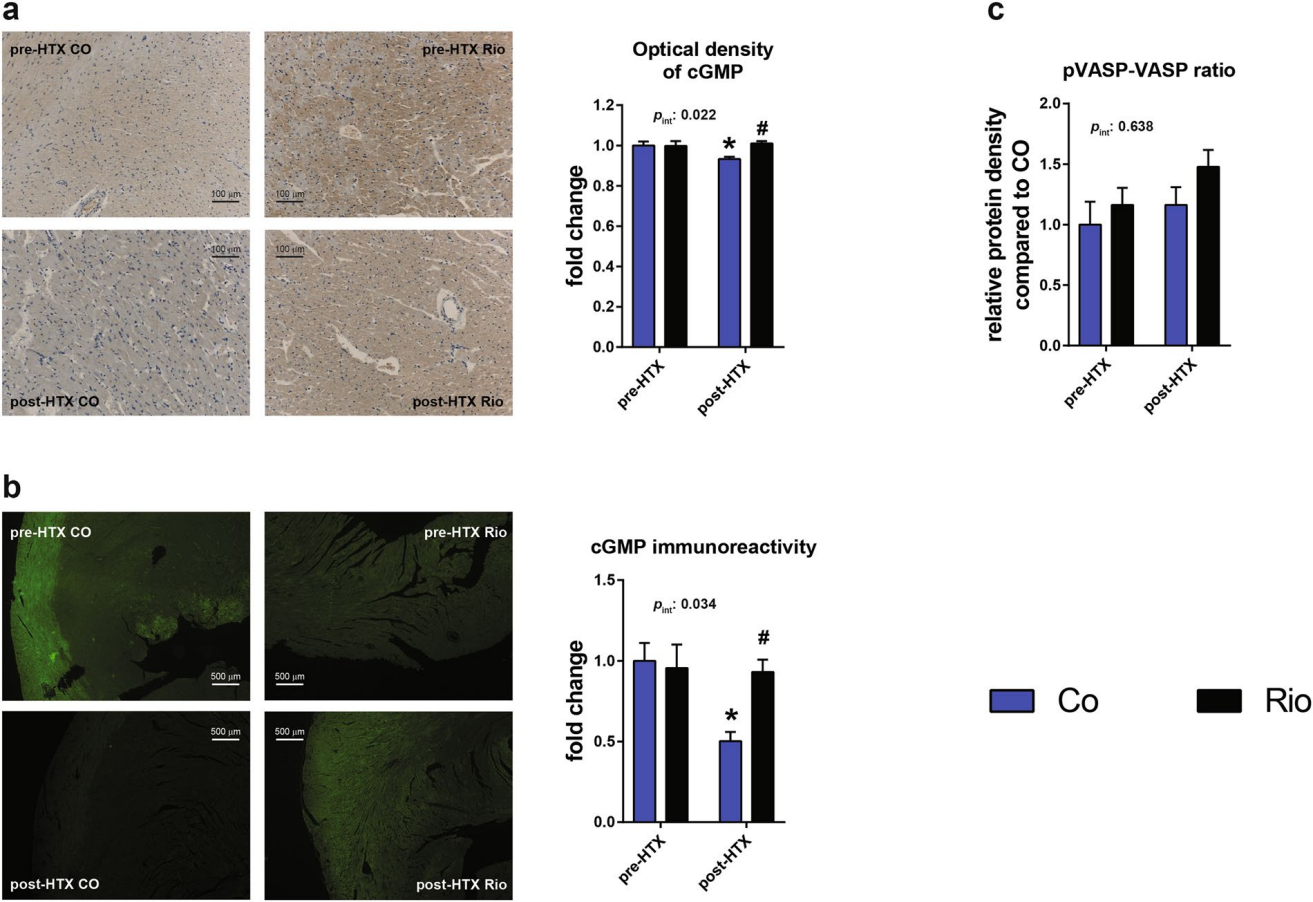
Riociguat increases cGMP content and thus cGMP-dependent signaling following transplantation. cGMP immunoreactivity (**a**,**b**) following transplantation was significantly decreased in COHTX myocardial samples, while riociguat treatment preceding transplantation significantly increased its amount. The elevation of myocardial cGMP led to increased protein kinase G activity, resulting in a trend towards elevation of VASP phosphorylation ratio (**c**). *p < 0.05 vs. CO, #p < 0.05 vs. COHTX; VASP: vasodilator-stimulated phosphoprotein; pVASP: phosphorylated VASP.

I/R injury during cold ischemia resulted in a significant decrease in myocardial cGMP content of COHTX hearts (Fig. 1). In contrast, preconditioning with riociguat prior to transplantation significantly increased cGMP immunoreactivity in the RioHTX group compared with their controls, respectively (Fig. 1). The elevation in tissue cGMP content resulted in a trend (p = 0.16) towards increased phosphorylation ratio of vasodilator-stimulated phosphoprotein (pVASP/VASP) in myocardial tissue homogenates of RioHTX hearts (Fig. 1), indicating increased PKG signaling in this group compared with COHTX.

### Antioxidant defense was reinforced in riociguat-treated transplanted hearts, decreasing the deleterious effects of nitro-oxidative stress

**Figure 2:**
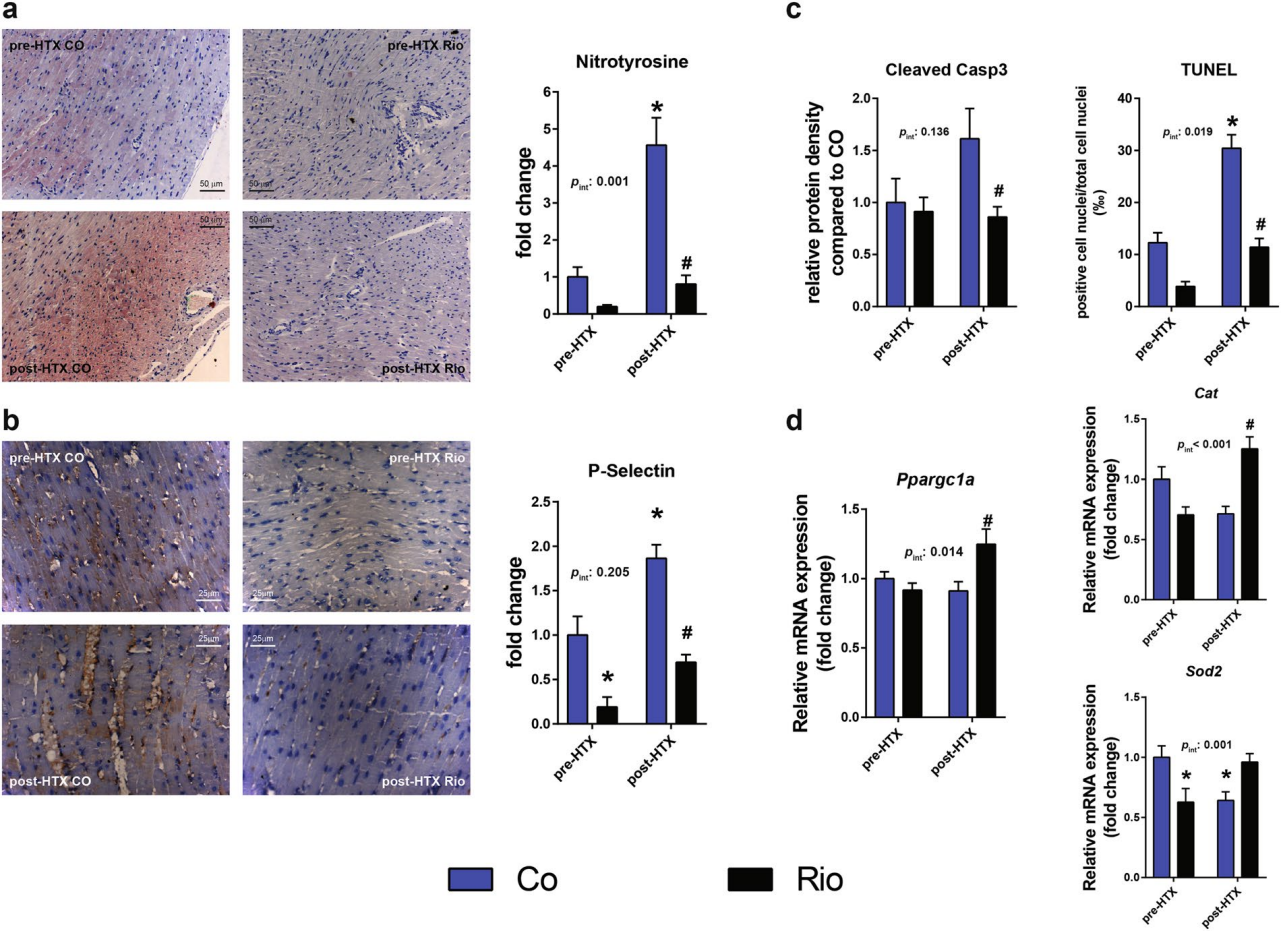
Deleterious effects of I/R injury are diminished via an increase in antioxidant capacity following riociguat preconditioning. Nitrotyrosine (**a**), as well as P-selectin (**b**) immunoreactivity following transplantation was significantly elevated in COHTX myocardial samples, resulting in an increase in DNA strand-breaks, thus apoptosis, as confirmed by TUNEL staining (**c**). Apoptosis might also have been induced in these animals, as implied by a trend towards an increase in caspase 3 activation (**c**). All of these changes were normalized by riociguat (**a–c**), in the background of which reinforced antioxidant signaling might have been a significant component (**d**). *p < 0.05 vs. CO, #p < 0.05 vs. COHTX; Casp3: caspase 3; Cat: catalase; Ppargc1a: peroxisome proliferator-activated receptor gamma coactivator 1-alpha; Sod2: superoxide dismutase 2; TUNEL: terminal d-UTP nick-end labeling.

3-nitrotyrosine (3-NT) immunoreactivity of the LV myocardium reflects the severity of nitro-oxidative stress occurring during ischemia and reperfusion, which was significantly increased in COHTX hearts compared with CO (Fig. 2). The decreased cardioprotective effects of cGMP-mediated signaling and the parallel increase in oxidative stress resulted in a significantly increased number of terminal d-UTP nick-end labeling (TUNEL) positive cell nuclei in the myocardium of COHTX hearts after transplantation, implying pronounced DNA fragmentation in this group. Protein density of cleaved caspase 3, an indicator of apoptosis, was also increased in COHTX myocardial samples compared to RioHTX (Fig. 2). Neither nitrotyrosine content, nor the number of TUNEL positive cell nuclei differed significantly from CO in the RioHTX hearts, while both were significantly less in this group than in COHTX hearts (Fig. 2). Myocardial P-selectin immunoreactivity was also increased in COHTX samples reflecting enhanced platelet activation and leukocyte recruitment in the post-ischemic myocardium, while preconditioning with riociguat before transplantation effectively prevented this elevation as well (Fig. 2). Of note, riociguat also decreased P-selectin immunoreactivity in Rio samples compared to CO. Quantitative real-time polymerase chain reaction (qRT-PCR) measurements from myocardial samples showed that mRNA-expression of the master regulator of myocardial energy metabolism and inducer of antioxidant enzymes, peroxisome proliferator-activated receptor gamma coactivator 1-alpha (PGC1α) was significantly upregulated in the RioHTX group compared with COHTX (Fig. 2). Furthermore, significant changes in the expression of antioxidant enzymes superoxide dismutase (SOD-2) and catalase (Cat) was also observed, indicating reinforced antioxidant defense in RioHTX hearts (Fig. 2).

### Cardiac and coronary vascular function in the graft post-transplant were better following riociguat treatment compared with control

**Figure 3:**
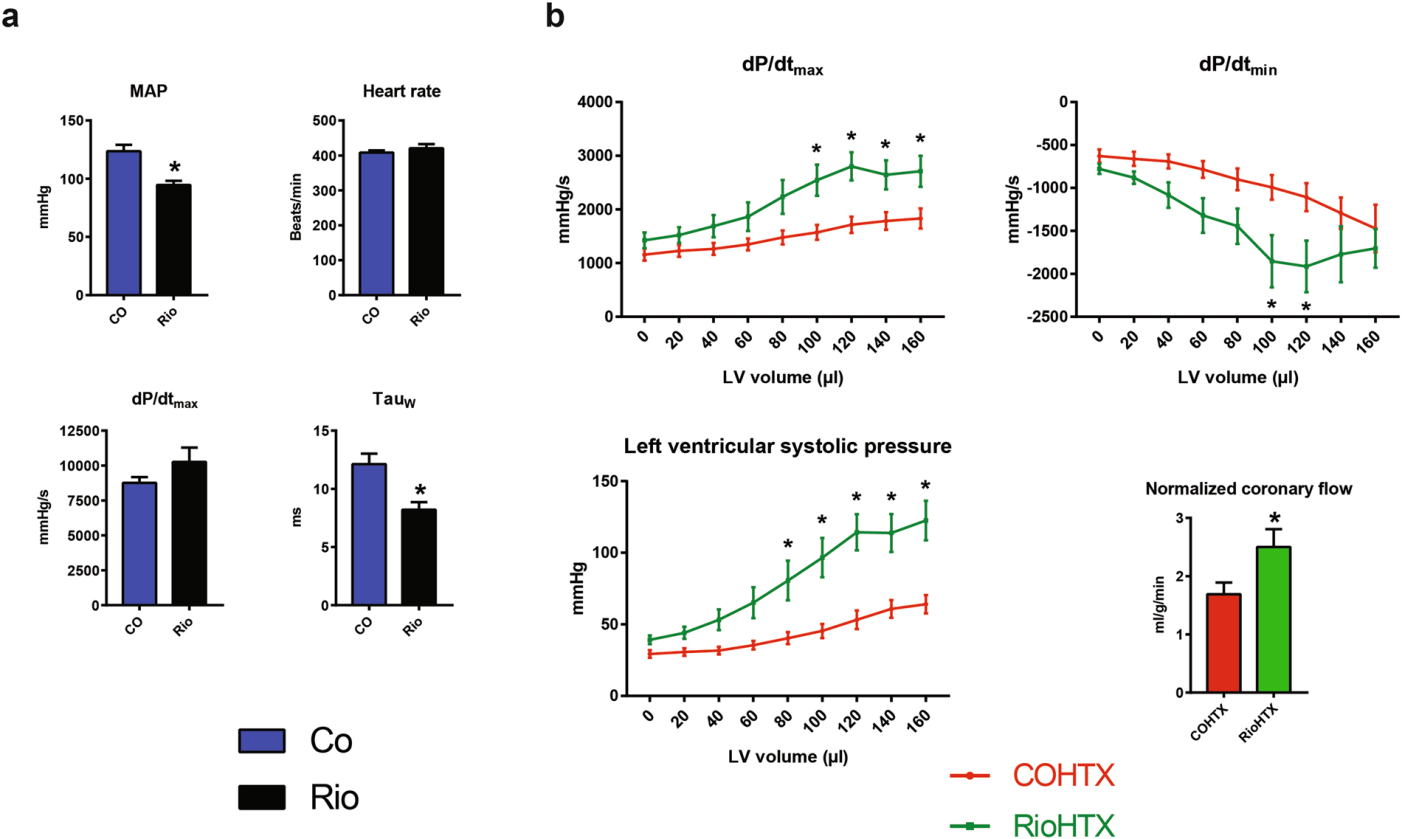
Systolic and diastolic function of riociguat-treated left ventricles are significantly improved after transplantation. Riociguat significantly improved active relaxation before transplantation, while decreased mean arterial pressure (**a**). Systolic pressures and contractility (**b**) at certain preload volumes were both significantly improved in RioHTX animals compared with COHTX rats. Active relaxation during diastole (**b**) showed a similar trend. A significant improvement in coronary flow (**b**) might have contributed to this effect. *p < 0.05 vs. CO, #p < 0.05 vs. COHTX; dP/dt_max_: maximal slope of pressure increment, dP/dt_min_: maximal slope of pressure decrement, MAP: mean arterial pressure.

Left ventricular systolic pressure (LVSP) and maximal slope of pressure increment (dP/dt_max_) measurements at gradually increasing left ventricular (LV) balloon volumes, which modeled increasing preload after transplantation, showed improved systolic function in the RioHTX group compared with COHTX (Fig. 3). Furthermore, preconditioning with riociguat resulted in an improvement in diastolic function at higher preload values, resulting in significantly increased maximal slope of pressure decrement (dP/dt_min_) compared with COHTX, suggesting improved myocardial relaxation (Fig. 3). Coronary blood flow was also significantly augmented after 1 hour of reperfusion in RioHTX hearts compared with COHTX group (Fig. 3), indicating significantly improved coronary endothelial function following riociguat preconditioning.

Preconditioning with riociguat resulted in decreased mean arterial blood pressure (MAP) and significantly improved active relaxation (tau-_W_) in Rio rats (Fig. 3), whereas the load-dependent contractile parameter dP/dt_max_ and heart rate were not affected (Fig. 3, Table 1).

**Table 1:** MAP: mean arterial pressure, BW: body weight, dP/dt_max_: maximal slope of pressure increment, dP/dt_min_: maximal slope of pressure decrement.

	Co	Rio	p
Systolic blood pressure (mmHg)	140 ± 8	110 ± 3	0.0042
Diastolic blood pressure (mmHg)	106 ± 4	81 ± 3	0.0007
MAP (mmHg)	123 ± 5	94 ± 3	0.0007
BW (g)	312 ± 2	315 ± 3	0.4993
Heart rate (bpm)	408 ± 6	420 ± 11	0.3832
dP/dt_max_ (mmHg/s)	8759 ± 414	10258 ± 1027	0.2007
dP/dt_min_ (mmHg/s)	−10069 ± 361	−10249 ± 940	0.8608

## Discussion

Our group presents evidence with the current findings that pharmacological preconditioning with the GC-1 stimulator riociguat ameliorates I/R injury during heart transplantation. This cardioprotective effect of riociguat manifested in improved coronary blood flow, as well as improved functional capacity of the LV of hearts preconditioned with the drug compared with placebo. Our results imply that the observed improvement in early post-transplant function following riociguat treatment is based on a significant increase in myocardial cGMP content and thus increased cGMP-dependent cardioprotective signaling, which results in decreased myocardial oxidative, as well as nitrosative stress and apoptosis through reinforcement of antioxidant capacity. This I/R injury reducing effect of riociguat has been shown in a murine infarction model using LAD ligation as well^2^, in which smaller infarct size and better long term preservation of LV systolic function was found when the drug was administered at the onset of reperfusion. These findings also support that GC-1 stimulation might be a powerful therapeutic treatment strategy for preventing I/R injury^2^.

Stimulation of GC-1 with preconditioning with riociguat in our donor rats resulted in a significant increase in myocardial cGMP immunoreactivity following transplantation in RioHTX hearts compared with COHTX (Fig. 1a,b), confirming the efficacy of our treatment. More important is whether the excess cGMP produced in response to riociguat resulted in elevated activity of PKG, which might be confirmed by measuring phosphorylation ratio of its target VASP^17^. Indeed, a trend towards elevation of this ratio was found in the grafts preconditioned with riociguat (Fig. 1d), which might suggest increased PKG activity in RioHTX animals.

PGC1α is a member of a family of transcription coactivators and a master regulator of myocardial energy metabolism. It plays a central role in the regulation of mitochondrial oxidative energy homeostasis^18^ and in endogenous antioxidant signaling^19^. It is highly likely that PGC-1α is intimately involved in disorders such as obesity, diabetes, and cardiomyopathy^20^ as well, which is further supported by a recent investigation concluding PGC1α deficiency accelerates the development of heart failure in mice challenged by transverse aortic constriction^21^. PGC1α was also shown to increase the expression of mitochondrial antioxidant enzymes, which in turn reduce nitro-oxidative stress^22^. Importantly, augmented cGMP signaling and increased PGC1α expression has been shown to be linked in various cell types^23,24^, including vascular endothelial cells^25,26^, implying that GC-1 stimulation might have additional beneficial effects via enhancing antioxidant defense. In line with these observations, significantly increased PGC1α expression was accompanied by a significant increase in the mRNA expression of antioxidant enzymes catalase and SOD2 in hearts of RioHTX animals compared with COHTX (Fig. 2d). In case of oxidative stress, in oxidized state of GC-1 becomes desensitized to available NO, leading to a decrease in cGMP production necessary for activation of downstream proteins and pathways^27^. As a result, preconditioning with riociguat significantly ameliorated nitro-oxidative stress in the myocardium of RioHTX animals, as evidenced by a significant decrease in nitrotyrosine immunoreactivity (Fig. 2a) and TUNEL positive nuclei (Fig. 2b) in the myocardium of these animals compared with COHTX. Therefore, apoptosis within the myocardium following HTX was significantly ameliorated by riociguat. Furthermore, as the pro-apoptotic^28^ cleaved caspase 3 was found to be significantly decreased in RioHTX hearts, apoptosis might have been ameliorated in these grafts compared with COHTX as well (Fig. 2b). Altogether, riociguat appears to have increased the capacity of endogenous antioxidant mechanisms, thereby protecting the myocardium from I/R mediated oxidative stress.

Microvascular obstruction is considered to be another major manifestation of myocardial reperfusion injury, and is associated with enhanced leukocyte invasion^29^. The cell adhesion molecule P-selectin expressed on activated endothelial cells plays a pivotal role during I/R injury by enhancing leukocyte adhesion across the intimal layer and causing thrombocyte aggregation^30,31^. These pathological pathways results in microvascular obstruction and myocardial damage. GC-1 has been shown to play a key anti-inflammatory role by inhibiting P-selectin expression on endothelial cells and thus leukocyte recruitment, which could ameliorate inflammation^32,33^. I/R injury during and after transplantation resulted in a significantly increased myocardial P-selectin immunoreactivity in COHTX grafts compared with CO (Fig. 2c). In contrast, preconditioning with riociguat significantly decreased P-selectin immunoreactivity to a level that was comparable with non-operated groups (Fig. 2c). This effect of riociguat might also have contributed to the decrease in oxidative stress in RioHTX hearts by decreasing the number of activated leukocytes in the myocardium and thus diminishing the amount of ROS generated by their oxidative burst.

The beneficial effects of riociguat on the molecular and microscopic level manifested in a significant improvement in cardiac function in the RioHTX group compared with COHTX. Hearts of RioHTX animals generated significantly higher LV pressure than COHTX grafts (Fig. 3a), as well as showed improved contractility and active relaxation, especially at larger LV volumes (Fig. 3a) following transplantation. Furthermore, coronary blood flow was significantly higher in the RioHTX group compared with COHTX (Fig. 3a), which might have contributed to the improvement observed in both systolic and diastolic function in the early phase following reperfusion. As such, riociguat appears to preserve donor organs during, and facilitate recovery after ischemia, and its effects are present both in endothelial cells and cardiomyocytes.

As GC-1 activators failed in the clinical setting due to their significant effect on blood pressure, we decided to conduct hemodynamic measurements in the donor animals at the time of peak plasma concentration of riociguat (Fig. 3b). Preconditioning with riociguat resulted in significantly lower MAP and better active relaxation during diastole (Fig. 3b). The observed decrease in MAP, although statistically significant, might not result in a clinically significant alteration in the setting of heart transplantation, as donor blood pressure is pharmacologically maintained before explantation of the donor organ.

## Conclusions

Our results suggest that preconditioning with riociguat improves donor heart function following transplantation in an experimental model of heterotopic HTX. To the best of our knowledge, the experimental data herein is the first to date evaluating the cardioprotective effect of GC-1 stimulation during heterotopic rat heart transplantation. Our findings show that stimulation of the GC-1 enzyme with riociguat could be a promising option to reduce I/R injury and to decrease the incidence of primary graft failure.

### Limitations

Interpretation of the results presented herein has limitations. Firstly, I/R injury is a reversible phenomenon in this model due to the unloaded reperfusion of the graft, which results in a relatively fast recovery. This complicates showing differences between experimental groups after a certain time of reperfusion. Thus, it is accepted to measure hemodynamic relations after 60 minutes of reperfusion even though it is a shorter period than in clinical practice^14,34–38^. Secondly, dosing or the mode of administration of riociguat might be different in a clinical setup. Lastly, the change in VASP phosphorylation ratio in response to preconditioning with riociguat is only a trend in this study, which warrants further experimental investigation to validate the relationship between cGMP enhancement and cardioprotection in the I/R setting.

## Materials and Methods

Parts of this section have been described in our recent publication^14^. We refer to each methodological aspect in the text accordingly.

### Animals

All animals used in the experiments described herein received humane care in compliance with the “Principles of Laboratory Animal Care” formulated by the National Society for Medical Research and the “Guide for the Care and Use of Laboratory Animals” prepared by the Institute of Laboratory Animal Resources and used by the National Institutes of Health (NIH Publication No. 86–23, revised 1996). Furthermore, all procedures during the study were reviewed and approved by the Ethical Committee of Hungary for Animal Experimentation and all methods were performed in accordance with the relevant international guidelines and regulations. As described in our recent publication^14^, male Lewis rats (250–300 g; Charles River, Germany) were housed in a room at 22 ± 2 °C under 12-h light/dark cycles and were fed a standard laboratory rat diet and water *ad libitum*. The acclimatization of the rats took for at least 1 week before the experiments.

### Experimental groups

Rats were randomly divided into four groups as follows. Control (CO, n = 7): donor rats receiving methylcellulose vehicle without consequential transplantation; (2) riociguat-control (Rio, n = 7): donor rats receiving riociguat without consequential transplantation; (3) transplant-control (COHTX): donor rats (n = 9) receiving methylcellulose vehicle followed by transplantation of the hearts into the recipients (n = 9); and (4) riociguat+transplant (RioHTX): donor rats (n = 9) receiving riociguat followed by transplantation of the hearts into the recipients (n = 9). Donor rats were treated orally with vehicle or riociguat as described below, while recipient rats received no treatment before the operations.

### Drug application

Riociguat was purchased from Bayer HealthCare (Berlin, Germany). 10 mg/kg body weight (BW) riociguat suspended in 1% methylcellulose solution vehicle was administered via oral gavage for the treated groups 24 and 2 hours before explantation, altogether twice before the procedure. This dose and administration method was chosen considering pharmacokinetic and –dynamic properties^11^ of riociguat as well as results of previous experiments^12,13^.

### Rat model of heart transplantation

Heterotopic heart transplantation in our experiments was performed in isogenic Lewis rats in order to avoid organ rejection, as described previously^14^. Briefly, donor rats were anaesthetized with isoflurane and were heparinized (25000 IU intravenously, iv.). Following exposure of the heart using bilateral thoracotomy, cardiac arrest was induced by infusion of 50 ml of cold cardioplegic solution (Custodiol, 4 °C, Dr. Franz Köhler Chemie GmbH, Bensheim, Germany) into the coronaries via the aorta. Then, the superior and inferior vena cava and the pulmonary veins were tied en masse with a suture and the heart was excised together with the aortic arch, which allowed for measurement of the coronary blood flow (CBF) later during the experiments. Hearts were stored in cold Custodiol at 4 °C following excision. Recipient rats were anaesthetized with isoflurane, heparinized (400 IU/kg iv.), and their body temperature was maintained at 37 °C using a heating pad. Occlusion of approximately two-centimeter segments of the infrarenal aorta and the inferior vena cava was followed by anastomosis of the aorta and the pulmonary artery of the donor heart end to side to the abdominal aorta and the inferior vena cava of the recipient rat, respectively. The duration of cold storage and implantation was standardized at 1 h (ischemic period) to minimize variability between experiments. Heparin was antagonized with protamine (400 IU/kg iv.) following completion of the anastomoses and the occlusion was released. Reperfusion of the donor organ with blood *in situ* was allowed for 60 minutes.

### Functional measurements in the donor

We compared the effect of preconditioning with riociguat on the hemodynamic parameters of the donor heart before explantation as described earlier^14^. A 2 F microtip pressure microcatheter (SPR-838, Millar Instruments, Houston, TX, USA) was used to carry out invasive hemodynamic measurements^39,40^. Sampling rate for these measurements was set to 1000 samples/s using a pressure-volume (P-V) conductance system (MPVS-Ultra, Millar Instruments) and the PowerLab 16/30 data acquisition system (AD Instruments, Colorado Springs, CO, USA). Animals were anesthetized with 1–2% isoflurane, and were placed on heating pads to maintain core temperature at 37 °C. The left external jugular vein was cannulated with a polyethylene catheter for fluid administration. At initiation of the measurements, mean arterial blood pressure (MAP) and heart rate (HR) were recorded, which was followed by advancement of the catheter into the left ventricle (LV) under pressure control. We used only the pressure signal of the microcatheter to calculate the maximal slope of systolic pressure increment (dP/dt_max_) and diastolic pressure decrement (dP/dt_min_) with a special P-V analysis program (PVAN, Millar Instruments).

### Functional measurements in the graft

Cardiac function and coronary flow of the graft were measured as described earlier^14^. Briefly, after one hour of reperfusion, a 3 F latex balloon catheter (Edwards Lifesciences Corporation, Irvine, CA, USA) and the Millar micromanometer were introduced into the left ventricle via the apex. LVSP, dP/dt_max_ and dP/dt_min_ were measured at LV volumes 20–160 µl provided by the balloon catheter. The acquired data was used to construct LV pressure-volume relationships. CBF of the graft was measured by an ultrasonic flow meter (Transonic Systems Inc., Ithaca, NY, USA) mounted on the donor ascending aorta, the only outlet of which was through the coronaries; therefore, global coronary flow could be indirectly measured^14^.

### Tissue sampling from the donor

After completing P-V measurements in the recipient, donor hearts were excised and LV samples were taken. The samples were either snap frozen in liquid nitrogen and stored at −80 °C or were fixed in 4% buffered paraformaldehyde via immersion to perform the measurements described below.

### Quantitative real-time polymerase chain-reaction (qRT-PCR)

The myocardium samples were snap frozen after hemodynamic measurements and homogenized to extract total RNA as described previously^39^. Reverse transcription reaction (1 µg total RNA of each sample) was completed using the QuantiTect Reverse Transcription Kit (Qiagen), and the resulting cDNA samples were amplified on the StepOnePlus™ Real-Time PCR System (Applied Biosystems, Foster City, CA, USA) using TaqMan® Universal PCR MasterMix and TaqMan® Gene Expression Assays (Applied Biosystems) for the following targets: endogenous antioxidant enzymes superoxide dismutase (SOD-2; Rn00690587_g1) and catalase (Rn00560930_m1), and the master regulator of myocardial energy metabolism, the mitochondrial transcriptional coactivator peroxisome proliferator activated receptor γ coactivator 1α (PGC1α; Rn00580241_m1). Ribosomal Protein L27 (RPL27; Rn00821099_g1) was chosen as housekeeping for normalization of gene expression data^14^. The mRNA expression levels were calculated using the CT comparative method (2^−ΔCT^) and adjusted to a pool of CO group.

### Western blot analysis

LV tissue samples were homogenized, and protein concentration was determined as described previously^40,41^. Equal amounts of protein were then separated via gel-electrophoresis, and proteins were transferred to polyvinylidene fluoride (PVDF) membranes^40^. After blocking in non-fat milk, membranes were incubated with primary antibodies against vasodilator-stimulated phosphoprotein (VASP) and phospho-VASP (1:2000, Cell Signaling Technology, Danvers, MA, USA, #3112 and #3114) cleaved caspase-3 (1:2000, Cell Signaling Technology, #9662), followed by incubation in a horseradish peroxidase-conjugated secondary antibody. Immunoblots were developed by enhanced chemiluminescence detection, as described previously^40^. Protein band densities normalized to α-tubulin (1:10000, sigma T5168, Sigma-Aldrich, Budapest, Hungary) were quantified using Image Lab software (Bio-Rad, Hercules, CA, USA).

### Histology, immunhistochemistry

We fixed the left ventricular samples in buffered paraformaldehyde solution (4%), embedded in paraffin and 5-μm thick sections were cut. The nitro-oxidative stress marker nitrotyrosine was detected by immunohistochemical staining utilizing antibody #10189540 (Cayman Chemical, Ann Arbor, MI, USA) as previously described^14^ and nitrotyrosine positive area was analyzed with Image J software (NIH, Bethesda, MD, USA). For identification of P-selectin, immunostaining of paraffin-embedded sections was performed with the use of a mouse polyclonal antibody (#sc-8419, Santa Cruz Biotechnology, Dallas, TX, USA)^42^ and was analyzed also with Image J software (NIH, Bethesda, MD, USA). Intracellular cGMP content was visualized by immunohistochemistry utilizing #ab12416 (Abcam, Cambridge, UK), performed according to a previously described method^40^. For futher investigation of the myocardial cGMP content immunofluorescent labeling (Antibody: ab12416) was also performed. Terminal deoxynucleotidyl transferase-mediated dUTP nick end-labeling (TUNEL) reaction was performed (DeadEnd™ Colorimetric TUNEL System, Promega, Mannheim, Germany) to detect DNA strand breaks^14,40^.

### Statistical analysis

Data is expressed as mean ± SEM. *t*-testing, one- or two-way ANOVA followed by the appropriate post-hoc test were performed as appropriate. A mixed-effect ANOVA model was applied with Sidak’s post hoc test (correcting for multiple comparisons) to analyze the hemodynamic data at different LV volumes. *p* < 0.05 was considered statistically significant.

## Supplementary information

Supplementary Dataset 1.

## Data Availability

The datasets generated during and/or analyzed during the current study are available from the corresponding author on reasonable request.
