# Supplementary material for: Stimulation of soluble guanylate cyclase improves donor organ function in rat heart transplantation
Source: Sci Rep. 2020 Mar 24;10:5358. doi: 10.1038/s41598-020-62156-y (PMC7093516; doi:10.1038/s41598-020-62156-y)
Supplement: Supplementary file 1 — Supplementary Dataset 1. [file 41598_2020_62156_MOESM1_ESM.pdf]

Stimulation of soluble guanylate cyclase improves donor organ function in rat heart transplantation

Kálmán Benke MD PhD[1], Balázs Tamás Németh MD[1*], Alex Ali Sayour MD[1], Klára Aliz Stark MS[1], Attila Oláh MD PhD[1], Mihály Ruppert MD[1], Gábor Szabó MD PhD[2], Sevil Korkmaz-Icöz PhD[2], Eszter Mária Horváth MD PhD[3], Rita Benkő MD[3], István Hartyánszky MD PhD[1], Zoltán Szabolcs MD PhD[1], Béla Merkely MD PhD DSc[1], Tamás Radovits MD PhD[1]*

[1] Heart and Vascular Center, Semmelweis University, Budapest, Hungary; [2] Department of Cardiac Surgery, University of Heidelberg, Germany; [3] Department of Physiology, Semmelweis University, Budapest, Hungary

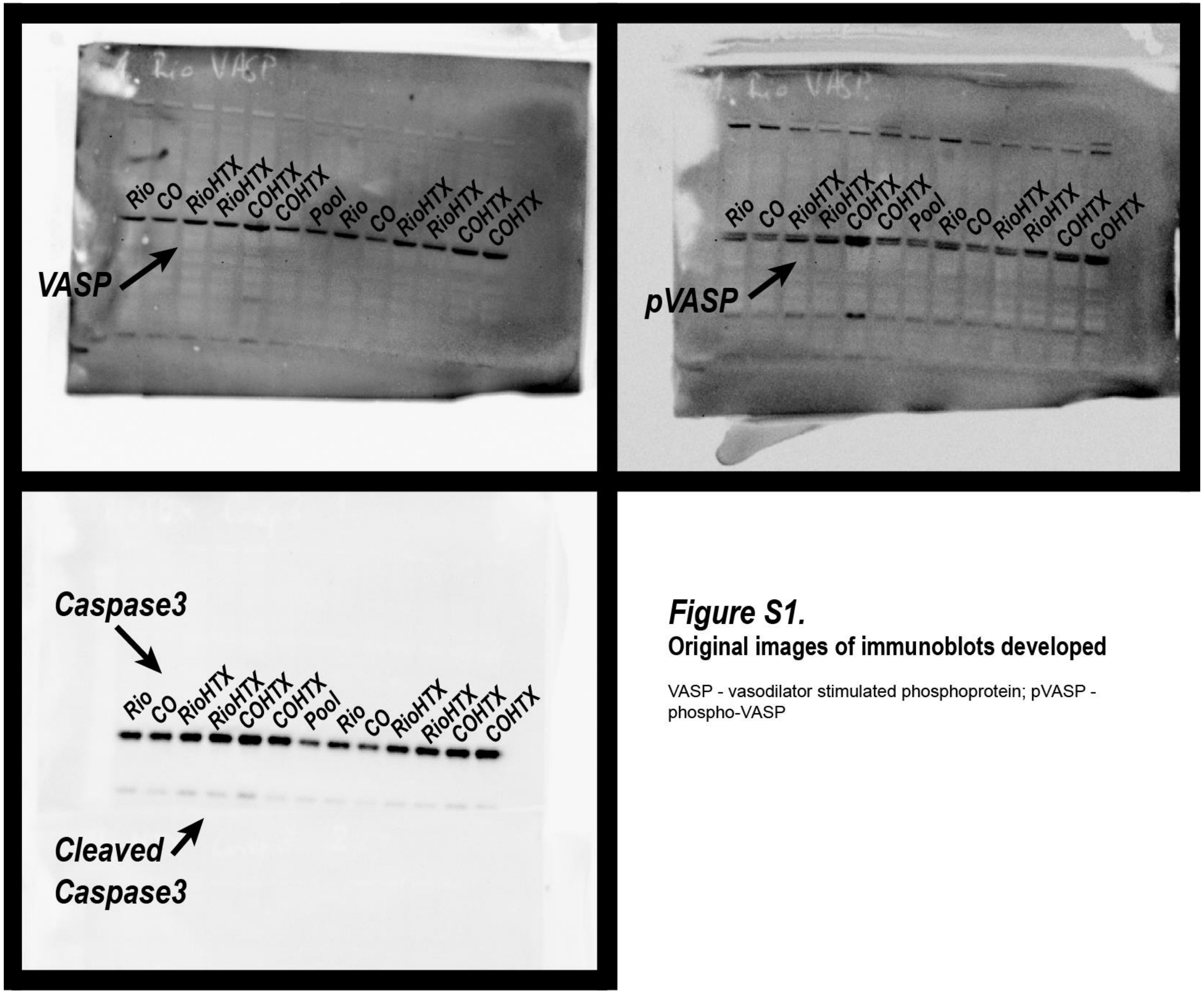

Figure S1.
Original images of immunoblots developed

VASP - vasodilator stimulated phosphoprotein; pVASP - phospho-VASP
